# Antioxidant Activity, Enzyme Inhibition Potentials, and Phytochemical Profiling of *Premna serratifolia* L. Leaf Extracts

**DOI:** 10.1155/2020/3436940

**Published:** 2020-09-24

**Authors:** Adelina Simamora, Adit W. Santoso, Kris H. Timotius, Ika Rahayu

**Affiliations:** ^1^Department of Biochemistry, Faculty of Medicine and Health Sciences, Krida Wacana Christian University, West Jakarta 11510, Indonesia; ^2^Centre for Enzyme in Health and Diseases, Krida Wacana Christian University, West Jakarta 11510, Indonesia

## Abstract

*Premna serratifolia*, commonly known as Arogo in Tentena-Sulawesi, is a popular vegetable. As a promising herbal tea and food ingredient, further investigation is required to find the best knowledge for medicinal use of *P. serratifolia* leaves. This research investigated the antioxidant activity of the ethanol (EEPS) and water (WEPS) extracts of *P. serratifolia* leaves, based on their scavenging activities on DPPH radicals and their reducing capacities (CuPRAC, total antioxidant/phosphomolybdenum, and ferric thiocyanate reducing power assays). The DNA-protecting effect by EEPS was tested using pBR322 plasmid DNA against •OH radical-induced damage. The inhibition potentials of both extracts against several enzymes related to metabolic diseases (*α*-glucosidase, *α*-amylase, xanthine oxidase, and protease) were evaluated. The phytochemical analysis was conducted by an LC-QTOF-MS/MS technique. EEPS proved to be a better antioxidant and had higher phenolic content compared to WEPS. EEPS demonstrated a protective effect on DNA with recovery percentage linearly correlated with EEPS concentrations. Strong inhibition on *α*-glucosidase and *α*-amylase was observed for EEPS; however, EEPS and WEPS showed weak inhibitions on xanthine oxidase and protease. LC-QTOF-MS/MS analysis identified seven main components in EEPS, namely scroside E, forsythoside A and forsythoside B, lavandulifolioside, diosmin, nobilin D, campneoside I, and isoacteoside. These components may be responsible for the observed enzymes inhibitions and antioxidant properties. *Premna serratifolia* leaves can be an appropriate choice for the development of nutraceutical and drug preparations.

## 1. Introduction

Metabolic diseases including type 2 diabetes mellitus (T2DM) and hyperuricemia have become major public health problems with increasing prevalence worldwide [1]. These diseases have been known to be related to the development of cardiovascular diseases. In each case, abnormally high enzyme activity is observed, such as *α*-glucosidase and xanthine oxidase activities for diabetes mellitus and gouty arthritis, respectively. Enzyme inhibition and the treatment for inflammation, as in the case in gouty arthritis, have become effective clinical approaches for the treatment of these diseases [2]. Synthetic inhibitors are the first-line drugs prescribed for the management of these disorders, such as acarbose for type 2 diabetes mellitus (T2DM), allopurinol for gouty arthritis, and sodium diclofenac for the treatment of inflammation. However, they reportedly cause unfavorable side effects. Acarbose has been associated with abdominal discomfort and hepatotoxicity [3]. Allopurinol also presents some adverse effects such as allergy, liver function abnormalities, and nephropathy [4]. Nonsteroidal anti-inflammatory drugs cause gastrointestinal and renal toxicities [5]. Therefore, it is of great priority to find new enzyme inhibitors that are more affordable and less toxic and have fewer side effects.

Plants have been a potential source for the discoveries of pharmacological active compounds including enzyme inhibitors that can be used as lead compounds for drug development. Plants can also be used as dietary adjunct to the existing therapy. Studies have evidenced that plant-based bioactive compounds demonstrated efficient inhibition to relevant enzymes associated with metabolic diseases [6, 7].

The species *Premna serratifolia* belongs to the family Lamiaceae. It is native to tropical and subtropical regions, including Indonesia, Australia, and India. The shrub plants can grow up to one and half meters. The leaves of *P. serratifolia* are commonly consumed as food ingredient in Tentena, Central Sulawesi, Indonesia, where it is known as Arogo. Different parts of *P. serratifolia* including fruit, roots, barks, and leaves have been used in folk medicine for the treatment of a number of illnesses, such as stomach disorders, diabetes, cough, rheumatism, inflammatory, and cardiovascular disorder [8]. Pharmacological studies conducted so far confirm on the ethnomedicinal uses of *P. serratifolia* including antioxidant, antiarthritis, antiparasitic, and cardio- and gastroprotective activities [9]. To date, studies conducted on *P. serratifolia* mainly involve roots, barks, woods, and stems from the plant. Only few papers have been published regarding the pharmacological properties of the leaves of *P. serratifolia*, such as tumor cell suppression activity [10], cytotoxic activities on neuroblastoma and melanoma cell lines [11], and anti-inflammatory and anticancer activities using animal model [12, 13]. Recently, we reported antidiabetic and antioxidant properties of the water extract of *P. serratifolia* leaves [14]. The present study aimed at extending this investigation on the antioxidant (reducing power and DNA protective) activities and enzyme (*α*-glucosidase, *α*-amylase, xanthine oxidase, and protease) inhibitory properties, of the water and ethanol extracts. Furthermore, the phytochemical profiles of the ethanol extract were also reported.

## 2. Materials and Methods

### 2.1. Chemicals

Acarbose, AlCl_3_ 6.H_2_O, allopurinol, porcine pancreatic *α*-amylase (EC 3.2.1.1), 2,2-diphenyl-1-picrylhydrazyl (DPPH), Folin-Ciocalteu reagent, *p*-nitrophenyl *α*-D-glucopyranoside (*p*-NPG), Na-diclofenac, orlistat, rutin, protease (EC 3.4.23.6), xanthine, and xanthine oxidase from bovine milk (EC 1.17.3.2) were obtained from Sigma-Aldrich (St. Louis, USA). PBR322 plasmid DNA was obtained from BioLabs (Boston, USA). Ammonium molybdate, NaNO_2_, and starch were purchased from Merck (Darmstadt, Germany). Gallic acid was purchased from Santa Cruz Biotechnology (Dallas, USA). All remaining reagents were of the highest purity available (>98%). All solvents were of analytical grade.

### 2.2. Plant Material and Extracts Preparation

The leaves of *P. serratifolia* were collected from Tentena, Poso, Central Sulawesi, Indonesia, in September 2018. A voucher specimen (KWL017) was deposited. The ethanol extract of *P. serratifolia* (EEPS) was prepared by macerating 50 g of powdered dried leaves in 500 mL ethanol at room temperature for 3 days in the dark. Following filtration, the solvent was dried under reduced pressure by a rotary evaporator (BUCHI Labortechnik AG, Switzerland). The obtained extract was kept at 4°C in darkness for further analysis. The water extract of *P. serratifolia* (WEPS) was prepared using 2 g of powdered dried leaves which was decocted in 200 mL of boiled deionized water. The mixture was continuously stirred, and the temperature was kept at 90°C to a final volume of 100 mL. After filtration, the clear filtrate was freeze-dried and kept at 4°C until used. The residue was used to prepare stock sample solution in ethanol for various analysis.

### 2.3. Total Phenolic Content (TPC)

The phenolic contents of EEPS and WEPS were determined using the Folin-Ciocalteu method as described previously [15]. Sample solution (0.5 mL) was added with 2.5 mL Folin-Ciocalteu reagent (10% *v*/*v*, aqueous solution) and left to stand for 10 mins. Sodium carbonate (Na_2_CO_3_) solution (75 g/L, 2.5 mL) was then added to neutralize the reaction mixture. After incubating for 2 h, the absorbance was read at 765 nm (Biochrom Libra S22, Cambridge, UK). Gallic acid (12.50–200 *μ*g/mL) was used to generate a standard curve.

Results were expressed as mg gallic acid equivalent (mgGAE)/g dried material.

### 2.4. Total Flavonoid Content (TFC)

The total flavonoid contents were determined based on an aluminium chloride colorimetric method as described previously [16]. The reaction mixture consisted of extract solution (0.5 mL), NaNO_2_ (5% *w*/*v*, 0.15 mL), AlCl_3_ (10% *w*/*v*, 0.15 mL), and NaOH (1 M, 2 mL). After incubating for 15 mins, the absorbance was taken at 510 nm. Rutin (6.40–1000 *μ*g/mL) was used to generate a standard curve. Results were expressed as mg rutin equivalent (mgRE)/gram dried material.

### 2.5. Antioxidant Activity Assays

#### 2.5.1. DPPH Radical Scavenging Assay

The effect of EEPS and WEPS on DPPH radicals was determined based on a method described previously [17]. Different concentrations of leaf extracts (3 mL) were prepared, and into each of these solutions was added DPPH solution in ethanol (1 mL). The mixture was vigorously vortexed, thereafter incubated at room temperature in the dark for 30 mins. The absorbance was read at 517 nm using a spectrophotometer. BHT and ascorbic acid were used as references, and ethanol was used as a negative control. The percentage of scavenging activity was calculated by the following formula:

(1) $$\begin{array}{c} \text{DPPH inhibition }\left(\%\right)=\frac{\text{A control}-\text{A sample}}{\text{A control}}\times 100 \end{array}$$

where A control and A sample are absorbances of negative control and sample, respectively. IC_50_ was calculated from the linear regression equation obtained from the plot of inhibition % against concentration.

#### 2.5.2. Cupric Ion Reducing Antioxidant Capacity (CuPRAC) Assay

The cupric ion reducing antioxidant capacity was determined according to a method described previously [18], with some modifications. Extract solution (0.5 mL) was added into a reaction mixture containing CuCl_2_ (10 mM, 1 mL), neocuproine in ethanol (7.5 mM, 1 mL), and NH_4_OAc buffer (1 M, 1 mL, pH 7.00). Water was added to make a final volume of 4.1 mL. After incubating for 30 mins, the absorbance was read at 450 nm. The absorbance was read against blank solution which contained all the solution except for the extract. Trolox (10–320 *μ*g/mL) was used to generate a standard curve. Extract activities were expressed as mg trolox equivalent (mgTE)/gram dried material.

#### 2.5.3. Total Antioxidant Assay

The total antioxidant activity of the extracts was determined by a phosphomolybdenum method described previously [19]. Phosphomolybdenum reagent consisted of ammonium molybdate (4 mM), sulfuric acid (0.6 M), and trisodium phosphate (28 mM). In a capped tube, 3 mL of this reagent was added with a sample solution (0.3 mL). The reaction mixture was incubated in water bath at 95°C for 90 mins. After cooling the samples at room temperature, the absorbance was read at 695 nm. The absorbance of blank solution was read by replacing the sample with water. Trolox (40–1000 *μ*g/mL) was used to generate a standard curve. The total antioxidant activity was expressed as mg trolox equivalent (mgTE)/gram dried material.

#### 2.5.4. Reducing Power Assay

The total antioxidant activity of the extracts was determined by the ferric thiocyanate method as described previously [20]. Sample solution in water (1 mL) was mixed with phosphate buffer pH 6.6 (0.2 M, 2.5 mL) and potassium ferric cyanide K_3_Fe(CN)_6_ (1% *w*/*v*, 2.5 mL). The mixture was incubated for 20 mins at 50°C. After cooling at room temperature, tricholoracetic acid (10% *w*/*v*, 2.5 mL) was added, and the mixture was centrifuged at 3000 rpm for 10 mins. The upper layer (2.5 mL) was taken out and mixed with water (2.5 mL) and FeCl_3_ (1% *w*/*v*, 0.5 mL). The absorbance was read at 700 nm. Ascorbic acid (1.56–100 *μ*g/mL) was used to generate a standard curve. Results was presented as mg ascorbic acid equivalent (AAE)/gram dried material.

#### 2.5.5. DNA Protection Assay

Protection against DNA damage by extracts was evaluated using agarose gel electrophoresis as described previously [21] with some modifications. This method was based on the ability of EEPS to protect the supercoiled pBR322 plasmid DNA from •OH radicals induced by Fenton reaction. The reaction system contained 1 *μ*L of plasmid DNA (0.5 *μ*g), 2 *μ*L of FeSO4 (1 mM), 2 *μ*L of H_2_O_2_ (1 mM), followed by the addition of 5 *μ*L of extract of different concentrations. The final volume was brought to 15 *μ*L by the addition of 5 *μ*L of phosphate buffer saline (PBS, 10 mM, pH 7.4). The reaction mixture was let to incubate at 37°C for 30 min. Thereafter, the reaction was terminated by the addition of 2 *μ*L of a loading buffer consisted of glycerol (50%, *v*/*v*), EDTA (40 mM), and bromophenol blue (0.05%). The mixture was then loaded on 1% agarose gel containing 0.5 *μ*g/mL ethidium bromide in Tris/acetate/EDTA gel buffer and electrophoresis was run for 90 min (80 V) on an electrophoresis instrument (the Gel Doc Azure). The plasmid DNA was visualised and photographed under ultraviolet light. Densitometric analysis was carried out for the quantification of recovery percentage using the ImageJ software (version 52, java 8).

### 2.6. *In Vitro* Enzyme Inhibition Assays

#### 2.6.1. *α*-Glucosidase Inhibition Assay

Inhibition activities on *α*-glucosidase by the extracts and acarbose (a positive control) were determined according to a reported method [17], using *α*-glucosidase from *Saccharomyces cerevisiae* (EC 3.2.2.20) and *p*-nitrophenyl-*α*-D-glucopyranoside (*p*NPG) as a substrate. In brief, 50 *μ*L of sample solution of different concentrations was mixed with phosphate buffer pH 6.8 (50 mM, 50 *μ*L). A solution of *α*-glucosidase solution (0.5 U/mL, 50 *μ*L) was added, and the reaction mixture was preincubated at 37°C for 5 mins. To start the reaction, *p*NPG (1 mM, 100 *μ*L) was added, and the reaction mixture was further incubated at 37°C for 20 mins. The reaction was terminated by the addition of Na_2_CO_3_ (100 mM, 750 *μ*L), and the absorbance was read at 405 nm. Acarbose was used as a positive control. The percentage of inhibitions were calculated by the following formula:

(2) $$\begin{array}{c} \alpha -\text{Glucosidase inhibition }\left(\%\right)=\frac{\text{A control}-\text{A sample}}{\text{A control}}\times 100 \end{array}$$

where A control and A sample are absorbances of negative control and sample, respectively. IC_50_ was calculated from the linear regression equation obtained from the plot of inhibition % against concentration.

#### 2.6.2. *α*-Amylase Inhibition Assay

The inhibition activity of the extracts on *α*-amylase was determined according to the method described previously [22], with some modifications. Extract of different concentrations (100 *μ*L) was mixed 100 *μ*L of phosphate buffer pH 6.9 (200 mM, with 6 mM NaCl) and pancreatic porcine *α*-amylase (0.5 mg/mL, 100 *μ*L). The reaction mixture was preincubated at 37°C for 5 mins. To start the reaction, 200 *μ*L of starch solution (1%, *w*/*v* in phosphate buffer) was added, and the reaction mixture was further incubated for 10 mins. The reaction was terminated by the addition of 500 *μ*L of dinitrosalicylic acid color reagent. The test tubes were then incubated in a boiling water bath for 5 mins and diluted with water (14 mL). The absorbance was read at 540 nm. The absorbance of blank solution was read by replacing the enzyme with phosphate buffer. The percentage of inhibitions was calculated using the following equation:

(3) $$\begin{array}{c} \alpha -\text{Amylase inhibition }\left(\%\right)=\frac{\text{A control}-\text{A sample}}{\text{A control}}\times 100 \end{array}$$

where A control and A sample are absorbances of negative control and sample, respectively. IC_50_ was calculated from the linear regression equation obtained from the plot of inhibition % against concentration.

#### 2.6.3. Xanthine Oxidase (XO) Inhibition Assay

Inhibition activity of the extracts on xanthine oxidase (XO) was determined based on the method reported previously [23] with modifications, using porcine pancreatic xanthine oxidase and xanthine as a substrate. Sample of different concentrations (100 *μ*L) was mixed with xanthine oxidase (0.2 U/mL, 100 *μ*L). The reaction mixture was added with phosphate buffer pH 7.4 (50 mM, with 400 *μ*L) and preincubated at 37°C for 5 mins. The reaction was started by the addition of xanthine (0.3 mM, 200 *μ*L) and further incubated at 37°C for 30 mins. Thereafter, HCl (100 mM, 200 *μ*L) was added to stop the reaction, and the absorbance was measured at 290 nm. The absorbance was read against blank solution in which the enzyme was replaced by phosphate buffer. Allopurinol was used as a positive control, and buffer was used as a negative control. The percentage of inhibitions was calculated by the following formula:

(4) $$\begin{array}{c} \text{Xanthine oxidase inhibition }\left(\%\right)=\frac{\text{A control}-\text{A sample}}{\text{A control}}\times 100 \end{array}$$

where A control and A sample are absorbances of negative control and sample, respectively. IC_50_ was calculated from the linear regression equation obtained from the plot of inhibition % against concentration.

#### 2.6.4. Protease Inhibition Assay

Inhibition activity of the extracts on protease was determined using a modified method described previously [24], using azocasein as a substrate and protease. The reaction mixture consisted of azocasein (5 mg/mL, 100 *μ*L), protease (0.08 U/mL, 50 *μ*L), and extracts of various concentrations (50 *μ*L), and the mixture was incubated for 90 mins at 37°C. Thereafter, TCA (5% *w*/*v*, 400 *μ*L) was added, and the mixture was centrifuged at 15,000 rpm for 10 mins. The supernatant was added with NaOH (0.56 M, 700 *μ*L). The absorbance was read at 442 nm. Sodium diclofenac was used as a positive control, and buffer solution was used as a negative control. The percentage of inhibitions was calculated by the following formula:

(5) $$\begin{array}{c} \text{Protease inhibition }\left(\%\right)=\frac{\text{A control}-\text{A sample}}{\text{A control}}\times 100 \end{array}$$

where A control and A sample are absorbances of negative control and sample, respectively. IC_50_ was calculated from the linear regression equation obtained from the plot of inhibition % against concentration.

### 2.7. LC-QTOF-MS/MS Analysis

EEPS was subjected to an LC-QTOF-MS/MS analysis using an Acquity UPLC system (Waters Corp., Milford, MA, USA), coupled with an MS/MS detector of XEVO G2-S QTOF model. The chromatographic separation was carried out using a C18 column. The eluent gradient was consisted of mobile phase A (0.1% formic acid in acetonitrile) and mobile phase B (0.1% formic acid in water). The solvent flow rate was maintained at 0.6 mL/min at 40°C. A QTOF mass spectrophotometer equipped with an electrospray ionization (ESI) source was used to perform the MS analysis, using positive and negative modes. The MS conditions were as follows. The acquisition range was 50 to 1200 Da, and the source temperature was 120°C. The capillary voltage was at 2.0 kV, and cone voltage at 40 V. The collision energy varied between 15 and 40 V. The desolvation gas flow was maintained at 1000 L/h, and the desolvation temperature at 550°C.

### 2.8. Statistical Analysis

All experiments were conducted in three repeats. Results were presented as mean ± standard deviation (SD). Students' *t*-test and one-way ANOVA (SPSS software version 23 for Windows) were used to analyse the difference, and *p* < 0.05 was considered to be statistically significant.

## 3. Results and Discussion

### 3.1. Total Phenolic (TPC) and Total Flavonoid Contents (TFC)

The total phenolic and flavonoid contents of EEPS and WEPS are presented in Table 1. A significant difference in TPC (*p* < 0.05) was observed between EEPS and WEPS, with EEPS showing a more concentrated phenolics, approximately 7–8 folds, than WEPS. However, WEPS was observed to have higher content in flavonoids compared to EEPS (*p* < 0.05). Plant-based phenolics and flavonoids have been associated with medicinal properties. Extraction of these metabolites from plant preparations is known to be influenced by solvent polarity, as also observed in the present study. Results indicate that ethanol, a less polar solvent than water, was a more effective extractant for phenolic compounds recovery from the *P. serratifolia* leaves. Some earlier reports indicated similar pattern, in which ethanol extracts contained higher phenolics and lower flavonoids when compared with water extracts [25]. This could be due to the solubility difference of various heterogeneous structures of phenolic and flavonoid compounds in each extract. More specifically, several phenylethanoid glycosides (PhGs), which were identified by the LC-QTOF-MS/MS analysis (part 3.4, Table 3), were abundant in polyphenolic structures, such as isoacteoside, forsythoside A and B, lavandulifolioside, scroside E, and campneoside I. PhGs were known to be soluble in polar solvents (water) due to their glycoside substituents. Furthermore, the LC-QTOF-MS/MS also identified diosmin, a flavon glycoside. It is also worth pointing that a significant amount of phenolics and flavonoids present in the water extract suggests its application in domestic context.

### 3.2. Antioxidant Activities

Antioxidant activity of the extracts was evaluated using different methods which differ in terms of mechanisms and reaction conditions. EEPS and WEPS were determined for their radical scavenging activity based on DPPH assay and their reducing capacity (CUPRAC, phosphomolybdenum, and reducing power assays). DNA damage protection capacity was also evaluated as part of antioxidant activities of EEPS. Results are presented in Table 2.

EEPS and WEPS exhibited potent scavenging activities on DPPH radicals, with IC_50_ comparable to that of the standard ascorbic acid. Stronger activity was observed for EEPS than WEPS (*p* < 0.05). Scavenging activities found in these extracts are in agreement with previous studies [14, 26]. Previously, extract of other part of *P. serratifolia* has been reported to have good activity on DPPH radicals. Ethanol wood extract showed to have IC_50_ of 155 *μ*g/mL [27], which was weaker than ethanol leaf extract observed in the present study.

CUPRAC, phosphomolybdenum and reducing power assays were employed to evaluate the reducing potential of the extracts. The methods are based on the reduction of Cu(II) to Cu(I) in acidic condition, Mo(VI) to Mo(V), and Fe(III) to Fe(II), respectively. Higher values indicate higher reducing capacity. By all methods, both EEPS and WEPS showed reducing ability as can be seen in Table 2. However, EEPS consistently exhibited higher reducing activities compared to WEPS (*p* < 0.05), suggesting a stronger antioxidant activity. Antioxidant activities of various Premna species have been reported previously. *Premna corymbosa* and *Premna mucronata* previously showed to have reducing power capacity [28].

Previous studies have demonstrated that antioxidant activity of the plant extracts was strongly related to their phenolic content [15, 29]. This present work proved the antioxidant activity of *P. serratifolia* leaves, which derives from radical scavenging and reducing activities. It is worth noting that EEPS showed higher content in phenolics compared to WEPS. The difference in activity registered to EEPS and WEPS may be associated to the content of the phenolics in the extracts as observed previously (part 3.1). Furthermore, PhGs as identified in the ethanol extract by the LC-QTOF-MS/MS were rich in phenolic structures such as isoactoside, forsythoside A and B, lavandulifolioside, scroside E, and campneoside I. Phenolic compounds contain one or more hydroxyl moieties on the benzene ring. The acidic properties of the hydroxyl group and the nucleophilic property of the benzene rings are responsible for the antioxidant activity of the phenolics, possibly by donating a proton or an electron to scavenge free radicals or involving in reducing metal ions of higher oxidation number to lower number.

Oxidative stress is considered central in triggering pathological conditions in hyperglycemia and hyperuricemia. It has been reported that persistent hyperglycemia perpetuated reactive oxygen species (ROS) formation through various pathways, including advanced glycation end-product (AGE), polyol, and protein kinase C pathways [30]. In addition, catalytic oxidation of hypoxanthine to uric acid contributes to the ROS formation. It has been known that pancreatic *β*-cells are vulnerable to ROS-induced destruction [31]. Further, ROS has been associated with the progression of diabetic complications [32]. Therefore, enhancing cellular antioxidant ability through dietary or supplement intake is key for an effective treatment for T2DM and hyperuricemia.

**Figure 1:**
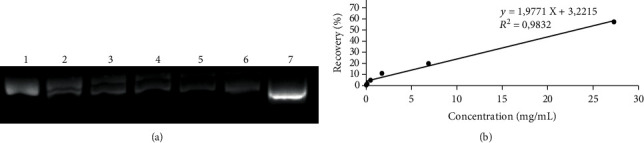
DNA protective effect of EEPS. (a) Lane 1: DNA + H_2_O_2_/Fe^2+^, lanes 2–6: DNA + H_2_O_2_/Fe^2+^ treated with EEPS (0.11, 0.43, 1.71, 6.83, and 27.33 mg/mL), lane 7: native DNA. (b) Concentration-dependent response of protective effect against DNA damage.

DNA protection effect was performed to evaluate the ability of EEPS to protect DNA from free radical-induced damage. The approach used in the present study was based on plasmid DNA breakage due to exposure to •OH radicals induced by Fenton reagent.  Figure 1 shows the electrophoretic pattern of DNA derived from pBR322 plasmid in the absence and presence of EEPS. Band on lane 7 shows pBR322 plasmid DNA in the absence of Fenton reagent, which was mostly in the supercoiled form with high electrophoretic mobility, as similarly observed in other studies [33]. Exposure of plasmid DNA to •OH radicals was shown to cause DNA strand breakage as indicated by open circular form with low mobility in electrophoreses (band on lane 1). Bands on lanes 2 to 6 were plasmid DNA treated with EEPS in increasing concentrations (0.11–27.33 mg/mL). Upon increasing addition of EEPS, DNA regained its native forms, as indicated by an increase in supercoiled form, thus suggesting the protective effect of the EEPS. Densitometric analysis further confirmed the recovery of the plasmid DNA due to EEPS addition. Positive linear correlation was observed between EEPS additions and DNA percentage of recovery (*R*^2^ = 0.9846), suggesting the significant role of the presence of EEPS in protecting DNA. The protective effect may be tentatively be ascribed to the polyphenolics in the extract, as has been shown also by previous studies [34]. Polyphenolics are known to be able to chelate metal ions such as Fe(II) and Cu(II) ions which participate in the formation of •OH radicals [35]. In particular, several phenylethanoid glycosides (PhGs) identified in this study contain polyphenolic structures that may be able to chelate these metal ions, such as isoactoside, forsythoside A and B, scroside E, and campneoside I. In fact, lavandulifolioside, which was identified in our extract, has been evidenced to exert protection effect on DNA *in vitro* [36].

### 3.3. Enzyme Inhibitory Activities

The antidiabetic potential of EEPS and WEPS were investigated based on their inhibition activities against *α*-glucosidase and *α*-amylase. Both enzymes are key in the digestion of polysaccharides, subsequently modulating postprandial blood glucose.

**Figure 2:**
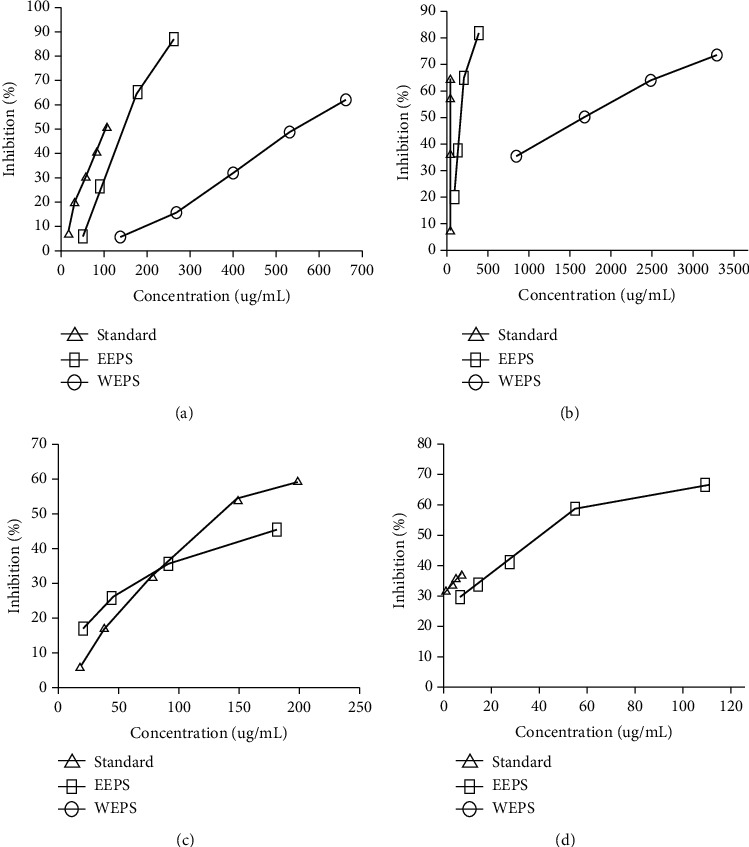
Concentration-response curves for the inhibition of EEPS, WEPS, and controls on (a) *α*-glucosidase, (b) *α*-amylase (c), xanthine oxidase, and (d) protease.

Both extracts were actively inhibited *α*-glucosidase, with inhibition percentages linearly correlated with concentrations (Figure 2(a)). However, significant difference in inhibition activity was observed between EEPS and WEPS (*p* < 0.05). The IC_50_ of EEPS and WEPS was 151.91 ± 4.80 and 558.15 ± 13.04 *μ*g/mL, indicating that EEPS had a stronger inhibition effect on *α*-glucosidase than WEPS. In the present study, acarbose was used as a reference, yielding IC_50_ of 100.38 ± 2.19 *μ*g/mL. These results suggest that acarbose had more potent inhibition activity than EEPS and WEPS. Acarbose, a prescribed antidiabetic agent, is known to act through inhibition on *α*-glucosidase in a competitive mode, similar to that of WEPS, as previously reported [17].

EEPS was observed to have a potent inhibitory activity on *α*-amylase compared to WEPS. As shown in Figure 2(b), the inhibitory activity increased with increasing EEPS concentrations, although a lower activity was obtained than acarbose (IC_50_ values of 201.31 ± 2.43 and 152.46 ± 8.43 *μ*g/mL, respectively). However, it was observed that WEPS had no inhibitory activity on *α*-amylase.

This study demonstrated that *P. serratifolia* leaves are inhibitors of *α*-glucosidase and *α*-amylase. This observation is in agreement with our previous study, in which it was shown that water extract inhibited *α*-glucosidase in a competitive mode [14]. On a previous report, the leaves of *P. serratifolia* have been reported to have hypoglycemic activity using animal model [37]. The present study indicates that the antidiabetic effect is probably mediated by its inhibition effect on *α*-glucosidase and *α*-amylase. Previous study on *P. serratifolia* using water extract has demonstrated close association between *α*-glucosidase inhibitory effect and total phenolic contents in the water extract [15]. PhGs, such as isoacteoside which was identified in our extract by mass spectrophotometry analysis, were previously reported to have hypoglycemic activity through its action on *α*-glucosidase [38]. In addition, isoacteoside was also shown to be able to inhibit the formation of AGE, which is a substance that can induce insulin resistance in adipocytes, hepatocytes, and muscle cells in mouse [39].

WEPS and EEPS showed a concentration-dependent activity on XO inhibition (Figure 2(c)). EEPS showed an appreciable inhibition activity on XO (IC_50_154.14 ± 4.62 *μ*g/mL), significantly stronger than WEPS (*p* < 0.05) which showed a modest activity on XO (IC_50_1688.07 ± 19.66 *μ*g/mL). However, the observed activity on EEPS is considerably weaker than allopurinol, a reference XO inhibitor, which displayed IC_50_ of 5.31 ± 0.49 *μ*g/mL.

Xanthine oxidase (XO) plays an important role in catalyzing the hydroxylation reaction of hypoxanthine to xanthine and ultimately to uric acid. An elevated level of blood uric acid (hyperuricemia) is pathological since it leads to the formation and deposition of monosodium urate crystal in the joints that causes gouty arthritis. In addition, the catalytic reaction also generates superoxide radicals, which contributes to oxidative stress condition. Lowering serum urate is an important approach in the management of hyperuricemia. Thus, inhibition on XO has become a therapeutic target in the treatment of hyperuricemia. The present study revealed that *P. serratifolia* leaf extracts demonstrated appreciable inhibition activities on XO. Previously, ethanolic wood extract of *P. serratifolia* was proven to have antiarthritis activity using animal model [40, 41]. However, to the extent of our knowledge, this is the first time XO activity of *P. serratifolia* leaves was reported. Finding in this study suggests that the leaf extracts of *P. serratifolia* are able to decelerate uric acid formation through inhibition on XO, thus can be beneficial in mitigating hyperuricemia and ROS generation. XO activity observed in EEPS and WEPS may be attributed to the phenolic compounds in the extracts, as have been shown by previous studies [42]. Several PhG compounds have been shown to have hypouricemia activity using hyperuricemic mice induced by potassium oxonate [43].

Proteases are involved in almost all biological processes in organisms, including metabolic and physiological processes, indicating their crucial role in maintaining homeostasis. Indeed, unregulated and excessive protease activity has been implicated in pathological conditions, such as in neurodegenerative, cancer, and autoimmune and inflammatory diseases [44]. Therefore, inhibition on proteases is a potential target on the development of therapeutic drugs. In the present study, EEPS and WEPS were screened for their inhibition activity on protease. As shown in Figure 2(d), EEPS showed inhibition activity against protease in a concentration-dependent manner (the IC_50_ of EEPS 60.54 ± 5.10 *μ*g/mL). However, the activity was observed to be much weaker compared to the positive control sodium diclofenac (IC_50_24.62 ± 0.34 *μ*g/mL), which was a nonsteroidal anti-inflammatory drug. Results in the present study indicate that *P. serratifolia* leaves are able to modulate protease activities thus can be further explored as a potential source for plant-based proteases inhibitors. To date, limited studies have been reported for plant-based protease inhibitors.

### 3.4. LC-QTOF-MS/MS Analysis of the Ethanol Extract

**Figure 3:**
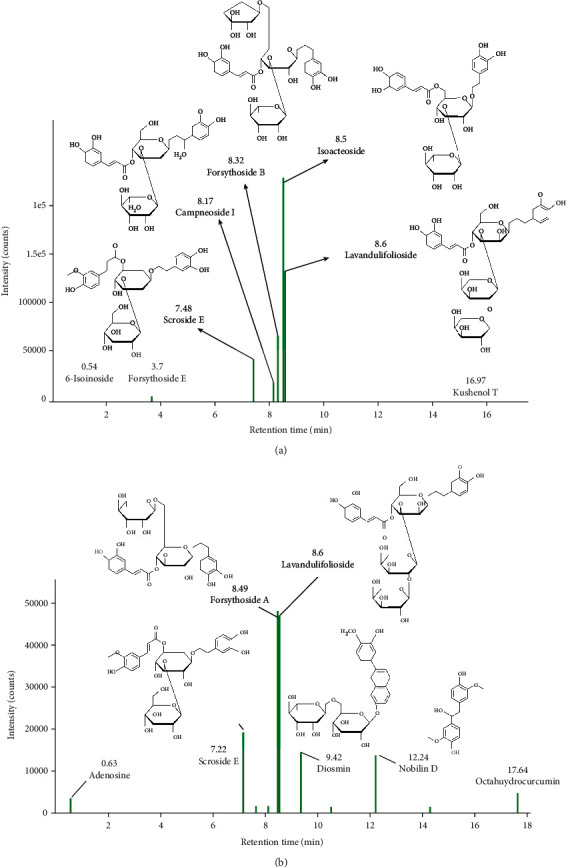
LC-QTOF-MS/MS chromatogram of major compounds identified in ethanol extract of *P. serratifolia* leaves using (a) negative and (b) positive modes.

Similar to the other members that belong to the family Lamiaceae, *P. serratifolia* is characterized by its phenylethanoid glycosides (PhGs). In fact, the main components identified in EEPS by LS-QTOF-MS/MS (Table 3) are of PhGs derivatives, i.e., isoactoside, forsythoside A and B, lavandulifolioside, scroside E, and campneoside I. Nobilin D is one of the major compounds in EEPS which is not of PhGs. Chromatogram of the LC-QTOF-MS/MS is shown in Figure 3. The PhGs have been studied for their multiple therapeutic potentials. It could be that PhGs identified in the leaf extract of *P. serratifolia* justify its therapeutic use in folk medicine.

Isoacteoside was the most dominant compound found in EEPS. Isoacteoside is a dihydroxypheynylethyl glycoside, which was also reported to exhibit multiple biological activities. Isoacteoside isolated from *Abeliophyllum distichum* was evidenced to have anti-inflammatory activity, by suppressing the production and expression of proinflammatory cytokines [45]. In addition, isoacteoside had the capacity to decrease amyloid-*β* deposition and cytotoxicity by preventing amyloid-*β* aggregation, suggesting its therapeutic potential for Alzheimer's diseases [46].

Forsythoside A (FTA) has been recognized to be responsible for multiple biological activities. Zeng et al. reported that FTA showed antiendotoxin or lipopolysaccharides (LPS) activity, exerting its anti-LPS effect by inhibiting the TLR4 signaling pathway [47]. LPS is one of bacterial products, and a high level of LPS in blood is associated with multiple organ dysfunctions and failures. Recently, FTA and campneoside I isolated from cell lines of *Syringa vulgaris* L. were evidenced to have good antioxidant activity, by inducing NRF2 protein which is a regulator of heme oxygenase 1 (HMOX1) [48]. Extract containing campneoside I showed antibacterial activity against *Staphylococcus aureus*, *Streptococcus pyogenes*, and *Streptococcus faecium*. From such antibacterial activity, the methoxy group of campneoside I was postulated to be the essential element for the antibacterial activity [49].

Forsythoside B (FTB) isolated from *Ballota nigra* showed antimicrobial activity against *Proteus mirabilis* and *Staphylococcus aureus* including one methicillin-resistant strain [50].

Lavandulifolioside was known to have cardiotonic pharmacological effects. In some *in vivo* studies, lavandulifolioside isolated from *Leonurus cardiaca* was evidenced to have antiarrhythmic effect [51]. Lavandulifolioside and Isoacteoside isolated from *Plantago lanceolata* L. reportedly had antispasmodic activity by inhibiting contractions induced by acetylcholine in animal model [52].

Diosmin is a flavone derivative which is also known as venosmine. In recent studies, anticancer activity was reported for diosmin against lung and breast cancer cell lines. The activity was enhanced by using diosmin-oxidovanadium (IV) complex compound [53]. Administration of diosmin to diabetic rats induced by streptozotocin proved to decrease the oxidative stress, as seen from the increase in antioxidant status of glutathione peroxidase, superoxide dismutase, and catalase levels [54].

It has been shown that nobilin D isolated from *Dendrobium nobile* exhibited better radical scavenging activities on DPPH and NO radicals, compared to vitamin C and resveratrol, in respective assays [55].

## 4. Conclusion

Taking all the above findings into consideration, we can come to the conclusion that EEPS and WEPS exhibited appreciable range of activities on antioxidant and inhibition on *α*-amylase and *α*-glucosidase. Both were not strong inhibitor for xanthine oxidase and protease. Therefore, EEPS and WEPS can be explored as a potential source of bioactive compounds for preventing metabolic syndromes. Phenylethanoid and flavone glycoside derivatives, which have various beneficial functions in health care, are the most detected compounds in ethanol extract from *P. serratifolia* leaves.

## Figures and Tables

**Table 1:** Total phenolic and flavonoid contents of *P. serratifolia* leaf extracts.

	Total phenolic content (mgGAE∗/g dried biomass)	Total flavonoid content (mgRE^†^/g dried biomass)
Ethanol	2.12 ± 0.06	9.43 ± 0.04
Water	0.27 ± 0.00	12.11 ± 0.20

∗mgGAE (mg gallic acid equivalent). ^†^mgRE (mg rutin equivalent). ^‡^Values expressed as mean ± SD (*n* = 3).

**Table 2:** Antioxidant activities of *P. serratifolia* leaf extracts.

	DPPH scavenging IC_50_ (*μ*g/mL)	CUPRAC mgTE∗/g dried biomass	Phosphomolybdenum mgTE∗/g dried biomass	Reducing power mgAAE^†^/g dried biomass
Ethanol	50.63 ± 0.93	27.61 ± 0.09	5.44 ± 0.03	1.43 ± 0.05
Water	66.83 ± 1.14	0.95 ± 0.15	0.51 ± 0.00	0.13 ± 0.00
Ascorbic acid	53.24 ± 0.82	NA	NA	NA

∗mgTE: mg/trolox equivalent. ^†^mgAAE: mg/ascorbic acid equivalent.

**Table 3:** Major compounds identified in the ethanol extract of *P. serratifolia* leaves using LC-QTOF-MS/MS based on positive and negative modes.

Compounds	RT	Area (%)	Formula	ESI	[M-H]^+^/^−^[M-H]^−^ (*mz*)	Major fragment
Scroside E	7.22	12.36	C_30_H_38_O_16_	+	673	672, 293, 147
	7.48	11.34		—	671	653
Campneoside I	8.17	4.45	C_30_H_38_O_16_	—	653	653
Forsythoside B	8.32	15.51	C_34_H_44_O_19_	—	756	755
Forsythoside A	8.49	31.33	C_29_H_36_O_15_	+	625	663, 642, 608, 325, 163
Isoacteoside	8.50	44.17	C_29_H_36_O_15_	—	623	623
Lavandulifolioside	8.60	22.75	C_34_H_44_O_19_	—	755	755
	8.60	30.57		+	757	774, 325
Diosmin	9.42	9.23	C_28_H_32_O_15_	+	609	609, 325, 463
Nobilin D	12.24	8.75	C_17_H_20_O_6_	+	321	307, 161

## Data Availability

All data used in the manuscript are already included in the manuscript.
